# Chronic pelvic pain treatment understanding what matters: a social media survey

**DOI:** 10.1530/RAF-24-0038

**Published:** 2025-01-10

**Authors:** Selina Johnson, Emma Evans, Dharani K Hapangama

**Affiliations:** ^1^ Walton Centre NHS Foundation Trust, Liverpool, UK; ^2^ Department of Women’s and Children’s Health, Institute of Life Course and Medical Sciences, University of Liverpool, Liverpool, UK; ^3^ Nuffield Department of Women’s and Reproductive Health, University of Oxford, Oxford, UK

**Keywords:** pelvic pain, treatment, questionnaire, survey, social media, understanding, chronic

## Abstract

**Abstract:**

Chronic pelvic pain (CPP) is a debilitating condition that reduces quality of life (QoL). In the United Kingdom, there is currently no standardised treatment pathway for women suffering from CPP. Therefore, it is essential to understand individuals’ concerns regarding CPP, their treatment experiences and what they seek from treatment. To do this, we conducted a two-month social media survey focused on the UK population to explore treatment experiences and identify the factors that people consider important to managing their condition. Of 1,279 respondents, women who completed ≥50% of the questions were included (*n* = 864; 68%). Results suggest that many women are living with moderate-intensity CPP and experience symptoms for 6 years (average) before receiving a diagnosis. Initially, most women see general practitioners and gynaecologists (90%), with varied care beyond these providers. Using an adapted STarT Back tool, 85% of respondents were classified as medium–high risk of poor outcomes based on physical, psychosocial, and psychological risk. Thematic analysis identified that people desire treatment validation/understanding, self-management, and support to manage pain and QoL. Notably, only 26% of respondents report satisfaction with their healthcare experience, suggesting that current treatment approaches do not address these themes. In conclusion, results suggest that treatment should focus on quality-of-life improvement to enhance CPP treatment outcomes and satisfaction. Findings endorse the need for improved and standardised treatment approaches that address patients’ needs.

**Lay summary:**

CPP is persistent pain in the lower abdomen or pelvis for at least 6 months. It is common and affects approximately 1 in 6 women in the UK. To improve treatment, it is important to understand people’s treatment experiences and treatment needs. We conducted a social media survey to understand how people with CPP experience treatment and what they would like from treatment. The survey was posted online for two months (May and June 2023) and received 897 responses. Responses suggested that people experience long waits before receiving help for their pain and that treatment journeys vary greatly. Overall, people reported low treatment satisfaction. People felt that effective treatment should improve pain and QoL. Themes of understanding their pain, knowing how to manage their pain and understanding treatments were identified as important. Clinicians should consider QoL and pain education as part of treatment.

## Introduction

Chronic pelvic pain (CPP) is defined as persistent pain in the lower abdomen or pelvis of a woman lasting at least 6 months in duration, not occurring exclusively with menstruation, intercourse or pregnancy (Engeler *et al.* 2024). The updated International Classification of Diseases (ICD-11) (adopted by the World Health Organization) considers all pains to be chronic after 3 months (Treede *et al.* 2019). This will likely lead to the revision of the current definition of CPP. CPP is a common condition in the United Kingdom (UK), with presentation and incident rates similar to asthma and back pain (Zondervan *et al.* 1999, Latthe *et al.* 2006). CPP can have a huge impact on how well someone can live and is associated with significant distress, disability and reduced quality of life (QoL) (Vitale *et al.* 2017, Della Corte *et al.* 2020). The annual medical costs for CPP in the UK are estimated to be £2,604 per woman. This is similar to or higher than the medical costs of other chronic diseases, such as heart disease and diabetes (Simoens *et al.* 2012). Indirect costs associated with loss of work productivity due to CPP are more than double this figure (£5,270 per woman per year) (Simoens *et al.* 2012).

CPP is understood to be influenced by complex pain mechanisms, and numerous treatment approaches are commonly utilised (Mardon *et al.* 2022). Treatment selection and pathways often vary depending on the identification of conditions/pathology that could be associated with CPP (e.g. endometriosis) and the availability of resources/specialists (Mardon *et al.* 2022). Many women experience significant delays in receiving care; for example, women in the UK wait, on average, 7–9 years before receiving a diagnosis of endometriosis (Jan *et al.* 2014, Ghai *et al.* 2020). Whilst various conditions, such as endometriosis, can be associated with CPP, tissue pathology is poorly correlated with the degree of pain and, for 40–55% of women with CPP, no pathology can be identified (Vincent & Evans 2021). Delays in receiving treatment/diagnosis and support to understand relevant pain mechanisms in the absence of pathology have been suggested to adversely affect the financial, physical and emotional impact of CPP (Cromeens *et al.* 2021).

To improve and better inform the management of CPP, it is important to understand how individuals experience this condition, its treatment and what they seek from treatment. Virtual communities and social media support groups are well utilised by patient groups. An online survey can canvas the opinions of these groups and reach people with many social demographic characteristics that can aid an understanding of treatment across social and geographic backgrounds (Wright 2005).

This study aimed to conduct a UK-based social media survey to understand i) treatments received and treatment experiences, ii) prominent concerns held by people with CPP and iii) what people desire and value as part of their treatment.

## Methods

An online anonymous survey (Supplementary material S1 (see section on Supplementary materials given at the end of the article)) was designed by a pelvic pain physiotherapist/researcher in conjunction with persons with CPP (*n* = 4) and a psychologist using Qualtrics XM software™ (Qualtrics, USA, https://www.qualtrics.com). Questions captured information concerning i) demographics (age, pain intensity (assessed using the numerical rating scale (1–10), duration of pain, time since diagnosis, ethnicity, residence and diagnosis), STarT Back tool (Hill *et al.* 2011) adapted for CPP (the tool is used as a screening tool to triage people to treatment based on physical, psychosocial and psychological risk factors; see Supplementary material S2)), ii) treatment history (clinicians seen, treatments received and treatment response), iii) treatment experience (people were asked to indicate whether they felt informed and part of choices about received treatment, if they had received helpful information and their satisfaction with care), iv) prominent concerns held by persons with CPP and v) what people desire/value (what is needed to understand the condition, important issues relating to treatment and how to measure effect). Questions included a mix of numerical, categorical and free-text responses (Questionnaire Supplementary material S1). A study flyer linked to the survey’s web address was posted on Facebook and X (formerly Twitter) social media platforms and promoted via UK-based charities, support groups and professional bodies (see Supplementary material S3) for a period of 2 months (1 May–30 June 2023).

The inclusion criteria for participation were as follows: i) patients with pain in the pelvis for 6 months or longer to ensure the responses reflected the experiences of those with CPP (Engeler *et al.* 2024), ii) adults and therefore only those aged 18 or over and iii) although CPP can affect all genders, considering the gender-associated differences in terms of experiences (Bartley & Fillingim 2013), we only included those who identified as female. The survey was beta-tested on four patients, from clinics at The Walton Centre NHS Foundation Trust, and the survey was modified according to their feedback. Patients were not reimbursed for their participation.

### Statistical analysis

Responses for persons who completed ≥50% of survey questions and resided in the UK or Ireland were included. All data were imported to Excel and then reviewed and cleaned by SJ as part of data familiarisation before coding.

#### Quantitative questions

Descriptive analysis was conducted using percentage and proportion ratios. For questions that asked people to provide numerical and categorical responses, mean/median responses were used to analyse data statistically. Spearman’s rank correlation was used to examine the relationship between age/ethnicity and time to diagnosis.

#### Open-ended questions

Where single-word responses were provided, descriptive analysis has been used. Manifest content analysis was used to capture the literal meaning of responses to treatment benefits and reflections. The frequency of responses was considered and organised into categories that reflected a pattern or shared meaning. To improve reliability, an ongoing discussion and engagement between SJ and AB (a clinical researcher experienced in reflexive thematic analysis (RTA) and pelvic pain) was used to develop depth and refine categories and meaning.

For questions that elicited longer more in-depth responses, such as future concerns and what people desire from treatment, RTA allowed the researcher experience to inform interpretations (Braun & Clarke 2006, 2019). SJ, a white female chronic pain physiotherapist specialising in CPP, conducted this. RTA involves six stages, including familiarisation with the data, generation of initial codes, development of themes, review of themes, defining and labelling themes and report of the analysis. A constructionist epistemology using a predominantly inductive approach was adopted to code data, considering both recurrences of concepts and their meaning/meaningfulness in the context of treatment, and themes were derived from repeated reading and interpretation of the data. Semantic and latent coding was utilised. Semantic (surface-level) coding dominated during the generation of initial themes, and latent coding was used to explore underlying meaning as themes were developed and reviewed. An experiential orientation to data interpretation was considered the most appropriate to highlight participants’ lived experiences and perspectives. Themes and subthemes were collaboratively discussed and reviewed (SJ and AB) to develop a richer understanding of data and construct final themes. Subgroup assessment of STarT classifications using broad theme titles was used to assess potential differences relating to future concerns and treatment desires.

## Results

### Participants

**Figure 1:**
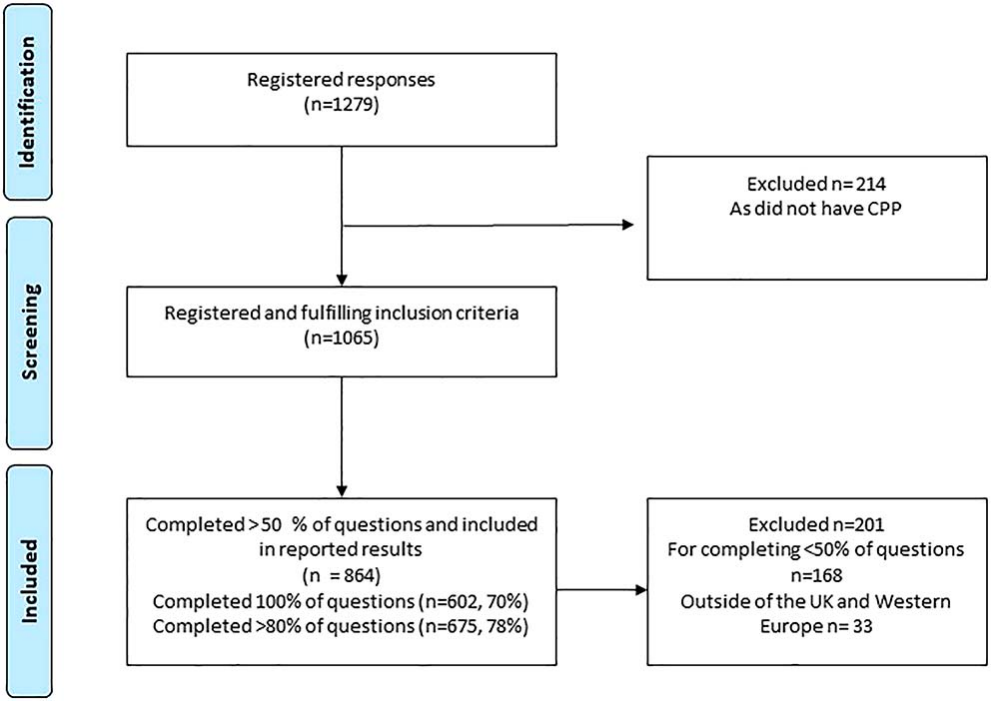
Flow chart of inclusion.

In total, 1,279 people registered on the survey, 1,065/1,279 (83%) fulfilled the inclusion criteria, and 864/1,279 (68%) were included in the data analysis (see Fig. 1).

### Demographics

Patient demographics are detailed in Table 1. Twenty persons stated that their country of residence was Western Europe and 14 of these stated Ireland as their area of residence whilst 6 left the area blank. As we could not exclude respondents from Ireland, these 6 were included. Median values, range and interquartile range (IQR) of responses are presented as data were not normally distributed. The median age of participants was 31, median years living with CPP was 10, and the median number of years since diagnosis and receiving support for their condition was 6. The median weekly pain intensity was 6/10. Nearly all participants (98%) were UK residents and had also been diagnosed with endometriosis (64%). No correlation between age (*r*^2^ = 0.03)/ethnicity (*r*^2^ = 0.09) and time to diagnosis was found.

**Table 1:** Demographics of participants (*n* = 864).

Characteristics	Values
Age (years)*	31 (16–74) (26–36)
Years with CPP*	10 (0.75–40) (5–15)
Years since diagnosis*	4 (0.08–36) (2–4)
Average weekly pain intensity^*^ (0–10)	6 (1–10) (5–7)
Ethnicity, *n* (%)	
White	797 (92%)
Mixed/multiple ethnic groups	26 (3%)
Asian/Asian British	27 (3%)
Black/African/Caribbean/Black British	9 (1%)
Other ethnic groups	5 (0.5%)
Residence	
United Kingdom	844 (98%)
Ireland/Western Europe	20 (2%)
Diagnosis	
Endometriosis/adenomyosis	557 (64%)
No diagnosis	129 (15%)
Bowel (IBS/IBD)	59 (7%)
PCOS	39 (5%)
Dysmenorrhea/menorrhagia	18 (2%)
Neuropathic pain	11 (1%)
BPS	12 (1%)
Others^†^	25 (3%)
No response	14 (2%)

CPP, chronic pelvic pain; IBS, irritable bowel syndrome; IBD, inflammatory bowel disease; PCOS, polycystic ovarian syndrome; BPS, bladder pain syndrome; PID, pelvic inflammatory disease; and FMS, fibromyalgia.

* Median, range, and interquartile range (IQR) of responses given as data were not normally distributed.

^†^ Vulvodynia, PID, FMS, fictitious, Mesh complication.

#### Adapted STarT Back tool

Out of 864 respondents, 834 (96%) completed the Adapted STarT Back tool. A median of 6 (IQR: 4–8) was reported, with a subscale score of 3 (IQR: 2–4); 15% of participants were scored as low risk (*n* = 124), 40% were scored as medium risk (*n* = 337), and 45% were scored as high risk (*n* = 373) (see Supplementary material S2 for a comparison of original and CPP modified version).

There were no major differences between STarT classification subgroups in the frequency of responses under broad theme headings (Supplementary material S4); however, the response language did differ. Evocative language was more commonly used to communicate distress by persons with medium/high scores compared to more explicative language in persons scoring low.

### Treatment history

#### Professional input

Participants were asked to select professionals they had seen about CPP. Responses (*n* = 840) indicated that they saw a variety of healthcare professionals (see Table 2), with most respondents (*n* = 827) seeing multiple professionals. When asked about the first 3 professionals they had seen about CPP (*n* = 757), 100% of cases reported seeing their GP and 90% of cases (*n* = 680) had seen a gynaecologist with varied care beyond these providers (Table 2).

**Table 2:** Healthcare professionals (HPs) seen in relation to chronic pelvic pain (CPP). Data are presented as *n* (%).

Professional	People seen by this HP (*n* = 840) for CPP	People seen by this HP within the first 3 HPs for CPP (*n* = 757)
GP	806 (96%)	757 (100%)
Gynaecologist	781 (93%)	713 (94%)
Urologist	184 (22%)	69 (9%)
Colorectal	227 (27%)	19 (2%)
Pain consultant	176 (21%)	42 (5%)
Physiotherapist	244 (29%)	78 (10%)
Chiropractor	84 (10%)	10 (1%)
Osteopath	84 (10%)	4 (0.5%)
Massage therapist	176 (21%)	1 (0.01%)
Psychologist	193 (23%)	26 (3%)
Pharmacist	361 (43%)	23 (3%)
Specialist nurse	260 (31%)	86 (11%)
Acupuncturist	134 (16%)	10 (0.01
None	13 (1.5%)	
Others	93 (11%)*	
Gastroenterologist		56 (7%)
A&E		33 (4%)
Radiographer		85 (11%)

* Nutritionist, reflexologist and surgeon.

#### Treatments received

Participants were asked to select received treatments and indicate if they had helped (tick box helped, not helped), if they had benefited from the treatment and how they had benefited (free text responses). Table 3 illustrates the findings (*n* = 788). Participants reported that seeing a specialist was beneficial in validating their condition and providing a diagnosis. Surgery often provided temporary pain reduction, but reduced efficacy was noted for repeat procedures. Medications were viewed to provide partial pain reduction, especially during flare-ups, but problems attributed to side effects were commonly noted. Hormonal treatments were reported to make symptoms less cyclical and more predictable. Psychology and physiotherapy were reported to help understand the condition and symptoms. Similarly, education was seen to improve understanding and increase perceptions of control (empowerment). Overall, many respondents reported benefit from a combination of strategies rather than a single treatment option.

**Table 3:** Treatments received (*n* = 788), treatment response and quotes associated with treatment benefits.

Treatment	% Received treatment	Response to treatment (%)		Type/frequency	Quotes associated with benefit
		Helped	Did not help		
Specialist referral	89%	56%	44%		‘Seeing a specialist and receiving education meant the start of the journey’
					‘Provided diagnoses’
					‘Condition recognised’
					‘Understand’
Surgery	66%	62%	38%	1 surgery = 48%, 1–3 surgeries = 26%, 3–5 surgeries = 20%, >5 surgeries = 7%	‘Surgery was helpful, only to be taken seriously, as diagnosis found’
					‘Surgery allowed me to be diagnosed with endometriosis’
					Overall: temporary pain reduction for varying periods of time with reduced efficacy when repeated
Medications	86%	63%	37%	Antidepressants = 50%, anticonvulsants = 10%, NAIDs = 64%, opioids = 57%, period-adjusting medications = 80%	‘Get through bad days’
					‘Make pain bearable’
					‘Edge off pain so can function but side effects’
					‘Help with flare-ups’
Hormonal	70%	41%	59%		‘Pain less cyclical and more spaced out’
					‘Control bleeding’
					‘More predictable & regulated’
Psychology	26%	54%	46%	Group CBT = 17%, counselling = 21%, couples therapy = 4%, EMDR/trauma therapy 6%, psychology 43%, psychosexual 4%	‘Therapy helps me navigate the fact I have to live with this and that there’s no relief, but that it’s not the end of the world’
					‘CBT helped me understand my symptoms better’
Exercise – physio	26%	57%	43%	Median = 1.5 courses, range = 1–22, IQR = 1–3	‘Pelvic floor muscles and try to make them less tight’
					‘Helped me to further understand my pain’
					‘Huge difference in helping me to understand how I hold my tension and pain, and what this means when I am in pain, I am far better equipped to deal with it’
Exercise	54%	57%	43%	Yoga = 59%, pilates = 32%, dilator/relaxation = 9%, gym including weights = 25%, stretches = 11%, swim 18%	‘Yoga/breathing/gym exercises have reduced bloating’
					‘Eased pain- yoga, swim, walk, gym’
					‘Helps with flare-ups’
					‘Helps with my energy and mental health’
Education	23%	62%	38%		‘Understanding what is going on with my body helps me to rationalise it and not spiral into fear as much (which makes the pain worse)’
					‘Manage flare-ups and function with flare-ups’
					‘Put me in charge - instead of surgeons’
					‘Manage pain and quality of life’
					‘Control’

CBT, cognitive behaviour therapy; EMDR, eye movement desensitisation and reprocessing; IQR, interquartile range; NSAIDs, non-steroidal anti inflammatory drugs.

### Treatment experiences

People were asked to indicate whether they felt informed and part of the treatment choices, if they had received helpful information and their satisfaction with care (*n* = 715).

Twenty-nine per cent of respondents felt fully informed about treatments they had received, 31% felt that healthcare professionals had listened to them, 47% felt part of treatment choices and decisions, and 29 and 28% had received information that had helped them to manage/understand CPP. Overall, fewer than 30% of participants reported satisfaction with the received care (26%) (Table 4).

**Table 4:** Reflections on treatments received.

Question	% Answering yes
Were you satisfied with the care provided by healthcare professionals?	26%
Did you feel fully informed about the treatment options you have received?	29%
Have you felt part of the choices/decisions regarding your care?	47%
Do you feel that the healthcare professionals you have seen have listened to you about your pain?	31%
Did you receive any information regarding your condition - that has helped you better manage/understand your pain?	28%

### Prominent concerns held by persons with CPP

#### Future concerns (*n* = 743)

Respondents’ answers regarding concerns about the future were categorised into the following three major themes: i) prognosis, ii) impact and QoL and iii) parenting and intimacy.

##### Prognosis

This title reflected feelings about CPP often being viewed as a progressive condition, ‘That this is just going to get worse, and the rest of my life will be miserable (ID 247 Endometriosis)’. Where individuals had reported not feeling listened to, many had developed a fear of things being missed in the future, ‘That I have cancer that’s undiagnosed … and not being taken seriously (ID 245 Endometriosis)’. Where respondents had received multiple and repeated interventions, statements illustrated increased pain anxiety and catastrophising and a bleaker outlook, ‘I will never get better, nothing can be done, I’ll never get my life back… no end in sight (ID 632 Menorrhagia)’ and ‘I feel there is little hope (ID 682 No identified condition)’.

##### QoL

Responses illustrated that CPP was negatively impacting QoL and there was a strong fear that this impact would grow with time, ‘Giving up things I love to do, as it gets worse and the older I get (ID 738 Endometriosis)’. Statements that signified the reduced ability to engage in valued activities were often written using self-deprecating language, illustrating a loss in perceived self-worth, ‘Unable to function at life (ID 793 Endometriosis)’. Additionally, there was a strong sense that people perceived living with CPP as a battle and questioned their resolve to continue the fight, ‘I’m afraid that it will continue to get worse and that I will get to a point where I won’t want to carry on (ID 628 IBS)’.

##### Parenting and intimacy

There were differences in responses based on the age of respondents. A strong sense of parental anxiety and relationship insecurity was evident. Older respondents (generally over 30) feared maintaining and losing roles, ‘being able to be a good parent (ID 40 Nerve pain)’ and ‘Will my partner and friends desert me (ID 595 Endometriosis)’. For younger respondents (generally under 30), there was a sense of thwarted opportunities and CPP derailing their future selves and aspirations, ‘Unable to have a romantic relationship, be normal (ID 8 Vulvodynia)’ and ‘Infertile, no life (ID 22 endometriosis)’. In younger respondents, whilst parenting was not a decided life goal, there was a strong sense of injustice that CPP would potentially remove this choice.

### What people desire and value as part of treatment

Three overarching themes were identified in response to what is important to understand about your condition and treatment (*n* = 607): i) validation/understanding, ii) management and iii) treatment.

#### Validation/understanding

Under this theme, two subthemes were identified (diagnosis and pain mechanisms).

#### Diagnosis

Under this subtheme, people described the importance of having a label. A diagnosis was viewed to support pain validation, ‘to help when dealing with other medical professionals (ID 94 Nerve pain)’, and enable access to treatment ‘so I can have a treatment plan (ID 46 No identified condition)’ and ‘Worthy of treatment (ID 272 Endometriosis)’. A diagnosis was seen to facilitate support, ‘That I am not alone, that people will listen to me (ID 216 No identified condition)’. People also desired to increase their knowledge and understanding. A diagnosis was viewed as a framework for this to empower future healthcare decisions, ‘It is very difficult to make decisions about your health when you don’t fully know what you have (ID 129 Bladder pain syndrome)’.

#### Pain mechanisms

Under this subtheme, people identified a need to understand what their pain is, why it persists, why nothing has worked from a biomedical approach and an explanation for the worsening of their pain. This was part of making sense of individual experiences and understanding how they could gain more control of their situation, ‘What is happening to me, and how I can manage it (ID 767 endometriosis)’. Many people had received multiple treatments with limited benefit, and there was a sense of distrust and that this could be avoided if information to support understanding could be improved, ‘It’s CHRONIC. there’s treatment but no cure (ID 624 no identified condition)’.

#### Management

Under this heading, two subthemes were identified (what I can do and support).

**What I can do **There was a strongly evident desire to reshape previous treatment experiences and become more self-autonomous with pain management by ‘Understanding triggers and how I can manage them (ID 604 Endometriosis)’ and ‘how to cope (ID 239 Nerve pain)’. Many responses indicated that people wanted treatment that supported ‘what can I do (ID 661 Endometriosis)’ and increasing self-efficacy. Respondents additionally indicated the wider context of pain needed to be appreciated as part of treatment with responses frequently highlighting mental health, family planning and flare-ups as important areas.

**Support** Support was needed to aid in the understanding of CPP by reducing feelings of alienation and normalising pain experience, ‘being able to speak to others (ID 618 No identified condition)’. Support was also discussed in terms of information sources. In the author’s experience, searching the Internet for information on health conditions can often heighten anxiety and uncertainty. These experiences appeared to be mirrored by responses that called for more widely available and reliable information/resources to support understanding, ‘not having to trawl the internet to find things myself (ID 373 Endometriosis)’. Workplace support and employers’ understanding was also commonly highlighted, ‘not having to prove to my boss my condition (ID 438 Endometriosis).’ Respondents additionally highlighted the importance of aftercare and support, ‘Aftercare following treatment, ….I have no clue about my life after (ID 528 Menorrhagia)’, with responses indicating that this was an area that needed to develop.

#### Treatments

Respondents wanted to understand treatment ‘options’ and feel ‘informed’.

**Options** Responses indicated that current information regarding different treatment options was viewed as inadequate. People wanted more information to understand potential ‘options available (ID 556 Endometriosis)’ and ‘Where I can go next (ID 38 Endometriosis)’. This was viewed to reflect beliefs (based on previous experiences) that their CPP has not been adequately understood or explored. Being left to live with CPP was viewed as damaging, and people wanted this to change, ‘I just want the next generation of girls to not wait so long for diagnosis and end up so damaged waiting for help (ID 564 PCOS)’.

**Informed** There was a clear desire for more detailed information and greater certainty on what treatments involve, the potential harms, ‘The risks and the evidence (ID 213 no identified condition)’ and any long-term risks, especially around fertility.

#### Who needs to understand (*n* = 607)

People reported that partners, friends and family, work and healthcare professionals needed to understand more about their condition. Although many reported their immediate social network was supportive, they also indicated a need to improve education and understanding of CPP. The desire to increase understanding was viewed to be shaped by previous negative healthcare experiences, feeling disbelieved, and improve people’s future healthcare experiences ‘very few have an understanding of it and its impact (ID 320 IBS)’. This was frequently highlighted when respondents discussed their GP or employers.

#### Measuring treatment efficacy (*n* = 565)

Whilst most respondents reported that they would know treatment was effective if it provided pain relief (58%), many reported that improved QoL was an important measure of treatment efficacy (40%) and 2% suggested that an effective treatment would be one in which they felt supported and listened to (Table 5).

**Table 5:** Measuring treatment efficacy – what is important. Data are presented as *n* (%) for a total of 533 responses.

Parameters for treatment efficacy	Values
Pain reduction	309 (58%)
Quality of life, activity and mental health	214 (40%)
Listened to/feel supported	9 (2%)

## Discussion

Survey results demonstrate that many women in the UK and Ireland are living with moderate-intensity CPP, and they wait, on average, 4 years before receiving a diagnosis or support. Initially, most were seen by general practitioners and gynaecologists (90%), with varied care beyond these providers. People received a variety of treatment approaches with varying efficacy. Reflections regarding treatment indicated low levels of satisfaction with care (26%), around one-third of participants felt adequately listened to (31%), and a similar proportion had received helpful pain management information (28%). Respondents defined effective treatment as pain reduction (58%) and improved QoL (40%). Thematic analysis identified that people desire validation and understanding to support their and others’ management of CPP, self-management strategies and informed treatment options.

Women had experienced pain for 4 years before receiving a diagnosis. Delays in diagnosis have been associated with variables such as age and ethnicity (Soliman *et al.* 2017). For example, studies have demonstrated a longer time to diagnosis/treatment for younger women compared to more mature women (Greene *et al.* 2009, Nnoaham *et al.* 2011). White ethnicity is negatively associated with time until diagnosis (Nnoaham *et al.* 2011, Soliman *et al.* 2017). Conversely, we found no association between age or ethnicity and time until diagnosis. This may reflect that the IQR (31, 26–36) for age was not sufficiently wide enough and that the sample was not diverse enough (92% white) to capture such associations. Explanations for treatment and diagnostic delays include social normalisation of pelvic pain and perceived illness validity (Greene *et al.* 2009, Soliman *et al.* 2017). These themes were identified as recurrent themes in the qualitative responses of the current survey.

Results additionally suggest varied care pathways beyond initial consultation with GP or gynaecologist. Further work is needed to understand current care delivery and reduce fragmented care and potential healthcare inequalities.

European Association of Urology (EAU) CPP guidelines highlight that biological, social and psychological factors all influence CPP and its management (Engeler *et al.* 2024). To quantify potential physical, psychosocial and psychological risk factors, we included the STarT Back tool (Hill *et al.* 2011), modified for CPP. This tool stratifies patients to treatment based on biopsychosocial impact and has been found clinically effective and cost-effective for low back pain (Saunders *et al.* 2022). The tool recommends physical therapy that includes pain science education (PSE) for those identified as medium risk and both physiotherapy and psychological interventions for those identified as high risk. Our results found that a large proportion of respondents (85%) were either medium or high risk, yet only 26% of respondents had received psychological treatment, 26% had received physiotherapy treatment, and 28% had received pain management education. What is not clear from the responses is whether the physiotherapy received was solely focused on pelvic floor dysfunction or was delivered in a psychologically informed way (i.e. additionally recognises the social and psychological factors that influence pain). Although the STarT Back tool is currently not validated in CPP, we suggest that a tool that supports stratification to appropriate treatments is needed in the context of identified variations in CPP care.

Overall, many people reported benefits not with one treatment on its own but with management involving a combination of strategies that helped them to manage different aspects/impacts of CPP. This highlights the complexity of CPP and further supports recommendations for multidisciplinary and multimodal management that address the variety of causative factors of CPP (Miller-Matero *et al.* 2016, Vincent & Evans 2021, Mardon *et al.* 2022). The need to consider a combination of strategies has also been highlighted by reviews examining the physiotherapy treatment of CPP, which found low-quality evidence for stand-alone treatments and the strongest evidence for interdisciplinary and multidisciplinary treatments (Fuentes-Márquez *et al.* 2019, Klotz *et al.* 2019, Leung-Wright 2020).

A shift in treatment focus from pain reduction to improving QoL has been associated with successful treatment of various chronic pain conditions (Katz 2002, British Pain Society (BPS) 2013). However, despite improving QoL being recognised as important to participants (42%), most participants (56%) felt pain reduction was the best measure of successful treatment. We assume that this may in part reflect the lack of awareness, as reported by participants, regarding different treatment approaches, how they work and how efficacy is measured. Improving education and awareness regarding different treatment approaches is recommended to improve patients’ and clinicians’ ability to make informed decisions regarding care and treatment expectations.

PSE is identified as a central component of successful pain management (Moseley & Butler 2015, Mardon *et al.* 2023). PSE aims to help people better understand their pain and explore better ways to manage it. In total, 28% of respondents reported receiving pain education, and those that did recive pain education reported that it helped increase control through supporting, understanding and knowledge ‘understanding my symptoms better and what they mean (ID 116 Endometriosis)’. PSE therefore represents an important aspect of care that our results suggest is not currently consistently explored or utilised.

A recent study exploring PSE for CPP has identified the following core themes: validation – a sensitised nervous system leads to over-protective pain, pain does not mean damage, how I think and feel can make it worse and I can change my pain… slowly (Mardon *et al.* 2023). In agreement, our survey also identified themes of validation/understanding – with a particular emphasis on what diagnosis, condition and symptoms mean, in addition to reassurance and honesty. Within the current survey, respondents reported feeling ‘fobbed off’ and ‘damaged waiting for help’. The need to improve condition and symptom validation is identified as an important theme in understanding CPP and treatment. We also noted a strong emphasis on understanding ‘what I can do’ as important. Our results complement recent PSE findings and highlight the importance of providing relevant information and self-management strategies to enable people to manage their pain.

Social support is also recognised as an important construct of how we understand and validate our healthcare experience (Karayannis *et al.* 2019). Social support has been found to have positive healthcare outcomes (Ahola Kohut *et al.* 2016, Matthias *et al.* 2016, Karayannis *et al.* 2019). Within the current survey, people reported desiring reliable information and support and struggling to locate support groups. Treatments and PSE could therefore be bolstered by considering the inclusion of support resources and social support signposting.

Current understanding suggests that treating pain intensity alone is not enough and CPP is best managed by a multidisciplinary team with the appropriate skills and understanding to address the variety of causative factors of CPP (Miller-Matero *et al.* 2016, Vincent & Evans 2021, Mardon *et al.* 2022). A shift in treatment focus from pain reduction to improving QoL has been associated with successful treatment of various chronic pain conditions (Katz 2002, British Pain Society (BPS) 2013). However, despite improving QoL being recognised as important to participants (42%), most participants (56%) felt pain reduction was the best measure of successful treatment. We assume this may in part reflect the lack of awareness, as reported by participants, regarding different treatment approaches, how they work and how efficacy is measured. Improving education and awareness regarding different treatment approaches is recommended to improve patients’ and clinicians’ ability to make informed decisions regarding care and treatment expectations.

To ensure that health care evolves in a way that aligns with the priorities and values of those it serves, it is essential to understand patients’ concerns. Survey respondents expressed worries about their prognosis, QoL and parenting, indicating that future treatments should focus on managing these areas effectively.

A strength of the current study was the large number of responses received, suggesting that CPP management is relevant to a large proportion of the population. An additional strength was that the survey was constructed by healthcare professionals, a psychologist with vast experience in survey design and persons living with CPP and it was beta-tested (*n* = 4) to ensure that it reflected important issues and avoided sources of bias commonly associated with survey design (Choi *et al.* 2005). There were, however, limitations that need to be considered. Although we observed a considerable response rate, the self-selected nature of the survey may reflect the inclusion of participants who were highly motivated by their CPP experience and limit the generalisability of findings. Additionally, as the response to questions was optional, we observed varying sample sizes for individual questions. This non-response bias limits how data are interpreted. We asked participants to retrospectively consider their treatment experiences, and therefore, we cannot exclude the influence of recall bias. The questionnaire was also only available via social media, which introduces administration bias, affecting the generalisability of findings as only persons who engage with social media could participate. We worked with patient advocate agencies and support groups to ensure that the survey had a wide and relevant reach.

## Conclusions

Results support the current understanding that many women with CPP experience delays in receiving treatment and that treatment journeys vary widely. Responses highlight that a focus on pain understanding and quality-of-life improvement could improve patient satisfaction and CPP treatment outcomes. Findings underscore the need for improved, standardised CPP treatment approaches in the UK healthcare system that address patients’ holistic needs and preferences.

## Supplementary materials



## Declaration of interest

SJ and EE have no competing interests. DKH is supported by the Wellbeing of Women (RG2137) and MRC (MR/V007238/1). DKH has received payment for presentations from Theramex and Gideon Richter.

## Funding

Throughout the review period, SJ’s research activity was supported by NHS research capacity funding and no other funding agency provided support for this review.

## Author contribution statement

SJ, HP and EE conceived the study. SJ administered the survey and led the analysis. HP and EE reviewed the results. SJ wrote the paper, and HP and EE contributed to the write-up and refinement of the submitted paper.
