# Supplementary material for: Chronic pelvic pain treatment understanding what matters: a social media survey
Source: Reprod Fertil. 2025 Jan 10;6(1):e240038. doi: 10.1530/RAF-24-0038 (PMC11795530; doi:10.1530/RAF-24-0038)
Supplement: Supplementary file 1 [file supplementary_materials.pdf]

Supplementary materials 1 (S1): Survey questionnaire

Title: Understanding what matters for people living with chronic pelvic pain- a social media questionnaire.

We are researching to try and understand the treatment experiences for women living with chronic pelvic pain (CPP). CPP is pain in the pelvic region that has been present for 6 months or longer, that is ***not*** associated exclusively with periods, intercourse, pregnancy, or is associated with recent childbirth/ or surgery in the abdomen (in the last 6 months).

By completing this 10–15-minute survey, you will be helping us to gain information that will inform and improve the delivery of future treatments.

If there are any questions you feel uncomfortable answering, then please leave these blank.

You will not be asked for any personal information that could be used to identify you. The answers you give will provide information regarding your pain and the treatments you have received. We will also ask you what you think could improve CPP treatment. Results from the survey will be shared via journal publication(s), and conference presentation. The results will be used to help improve future research that looks at CPP treatment.

All information collected will be kept until results have been shared via journal publication and conference presentation. After this time information will be stored according to the university's rules about storing information.

- ❑ Please tick box this box if you have read this information and you are happy to take part.

Thank you again for taking part in the study.

Background questions:

1. Do you have CPP, pain in the pelvis for 6 months or longer?
   Yes
   No
2. What is your Age
   (Free text)
3. Which gender do you identify with?
   Female
   Male
   Prefer not to say
4. Choose one option that best describes your ethnic group or background
   White
   a) English/Welsh/Scottish/Northern Irish/British
   b) Irish
   c) Gypsy or Irish Traveller
   d) Any other White background, please describe
   Mixed/Multiple ethnic groups
   e) White and Black Caribbean
   f) White and Black African
   g) White and Asian

h) Any other Mixed/Multiple ethnic background, please describe

Asian/Asian British

i) Indian
j) Pakistani
k) Bangladeshi
l) Chinese
m) Any other Asian background, please describe

Black/ African/Caribbean/Black British

n) African
o) Caribbean
p) Any other Black/African/Caribbean background, please describe

Other ethnic group

q) Arab
r) Any other ethnic group, please describe

5. What county do you live in? (Free text)
6. How many years have you suffered with chronic pelvic pain? (number)
7. When did you begin to receive investigations or treatment for your pelvic pain? (number of years ago).
8. Have any other names/ diagnosis been used to describe your symptoms other than chronic pelvic pain? (Free text)
9. Are you pre/peri/ post menopause? Please choose from the below options:
    a. having periods and no menopausal symptoms = premenopausal;
    b. having periods but menopausal symptoms = peri-menopausal
    c. Stopped having periods for >12 months and menopausal symptoms + menopausal
10. We would like to understand if giving birth has any influence on someone's pelvic pain and therefore, we would like to ask how many children you have been able to give birth to. (free text)

We would now like to ask you a few questions about your chronic pelvic pain.

Understanding your pain

1. What number best describes the average severity of your pelvic pain in a given week? (Please rate on a scale 0 -10 where 0= no pain at all to 10= the worst pain imaginable)
   0, 1,2,3,4,5,6,7,8,9,10
2. How much (on average) does your current chronic pelvic pain interfere with:

   No interference, slight interference, moderate interference, severe interference

   General activity
   Normal work (includes both work outside the home and housework)
   Walking ability
   Standing
   Sitting
   Sleep
   Social activities
   Mood
   Enjoyment of life

Relations with other people
Intimacy (sexual relationships)
Parenting / caring
Continence

3. We know pelvic pain can fluctuate and people can experience episodes of pain when their pain is especially bad, we would like to understand on average how often this happens for you.
   Categories: More than weekly, Weekly, Every few weeks, Monthly, Every few months, every 6 months, Yearly, never
4. When you experience a severe episode of pain what activities are you particularly unable to do? (Free text)

Modified Start back tool:

Thinking about the last 2 weeks please answer the following questions:

5. My pelvic pain has spread down my leg(s) at some time in the last 2 weeks

   Yes No

6. I have had pain in other parts of my body at some time in the last 2 weeks

   Yes No

7. I have only walked short distances because of my pelvic pain

   Yes No

8. In the last 2 weeks, I have dressed more slowly than usual because of pelvic pain

   Yes No

9. It's not really safe for a person with a condition like mine to be physically active

   Yes No

10. Worrying thoughts have been going through my mind a lot of the time

    Yes No

11. I feel that my pelvic pain is terrible and it's never going to get any better

    Yes No

12. In general, I have not enjoyed all the things I used to enjoy

    Yes No

13. Overall, how bothersome has your pelvic pain been in the last 2 weeks?

    Not at all Slightly Moderately Very Much Extremely

14. We know pelvic pain can fluctuate and people can experience episodes of pain when their pain is especially bad. We would like to understand on average how often this happens for you.

Weekly, few weeks, monthly, few months, 6 months, yearly, never

15. When you experience a severe episode of pain, what activities are you unable to do?

Treatments received.

1. Which of the following medical professionals have you seen in relation to your chronic pelvic pain? (Please select all that apply).
   GP
   Gynaecologist (a doctor who specialises in women's reproductive systems)
   Urologist (a doctor who specialises in conditions that affect the urinary tract and bladder)
   Colorectal specialist (a doctor who specialised in problems affecting the bowel)
   Pain consultant (a doctor who specialises in chronic pain)
   Physiotherapist
   Chiropractor
   Osteopath
   Massage therapist
   Psychologist
   Pharmacist
   Specialist nurse
   Acupuncturist
   None
   Other (please specify)

2. After seeing your GP, who were the first 3 health professionals you saw?
   1. free text
   2. free text
   3. free text

3. What types of treatment have you had to date? (Please select all that apply).
   Yes/ No response with linked logic connecting questions patients have answered yes to text box asking patients to define type and number received

   Specialist referral/ investigations *(for example laparoscopy (surgical exploration of abdomen), MRI scan, coloscopy (camera examination of the bowel), cystoscopy (camera examination of the bladder), ultrasound, urine or blood tests).*

   Injections (including botox, injections into the muscle or nerve blocks)

   Surgery If yes please state type (if known) and number you have undergone surgery (for example laparoscopic removal of endometriosis tissue, fibroid removal, hysterectomy, prolapse surgery etc)

   TENS

   Medications: Options for this: Antidepressants, anticonvulsants, NSIADS, Opioids, Period adjusting medications, Other:

   Hormonal treatments

Bladder instillations

Psychological therapies (e.g. CBT, ACT, group therapy, counselling).

Sexual counselling/ sex therapy

Pelvic floor manual therapy (e.g., deep tissue massage, stretching, dilator training etc.)

Pelvic Massage

Exercises provided by a physiotherapist If yes please sate the number of times you have seen a physiotherapist

Electrotherapies (e.g., Ultrasound, Laser, TENS, Interferential etc)

Neuromodulation (e.g., sacral nerve stimulator, intrathecal drug delivery system)

Acupuncture

Exercise: please specify exercise type e.g., Yoga / Pilates/ Meditation/ Relaxation exercises etc

Education provided by health professionals on pelvic pain (e.g., likelihood of recovery, things you can do to recover)

None

Other: Free text

4. Now thinking **only** about the times when your pain is especially bad, what treatments (from the ones you selected before) do you use? Tick to select

    Specialist referral/ investigations (for example laparoscopy, MRI scan, coloscopy, cystoscopy, ultrasound, urine or blood tests)

    Injections (including botox, injections into the muscle or nerve blocks) If yes please state type (if known) and number of injections you have received Type (Free text)
    Number received (number 0-20)

    Surgery (for example laparoscopic removal of endometriosis tissue, fibroid removal, hysterectomy, prolapse surgery etc)

    TENS unit

    Medications: Options for this: Antidepressants, anticonvulsants, NSIADS, Opioids, Period adjusting medications, Other:

Hormonal treatments

Bladder instillations

Psychological therapies

Sexual counselling/ sex therapy

Pelvic floor manual therapy – stretching, dilator training etc.

Massage

Exercises provided by a physiotherapist

Electrotherapies (e.g., Ultrasound, Laser, TENS, Interferential etc) if yes please state type

Neuromodulation (e.g., sacral nerve stimulator, intrathecal drug delivery system)

Acupuncture

Exercise e.g., Yoga / Pilates/ Meditation/ Relaxation exercises etc
Type (free text)

Education provided by health professionals on pelvic pain (e.g., likelihood of recovery, things you can do to recover)

None

Other: Free text)

Treatment experience

1. How have the treatments you have received helped you manage your pain? From the treatments you have listed, did they help?

   Categories for responses: tick box
   Treatment not received:
   Did help
   Did not help

   Specialist referral/ investigations
   Injections (including botox)
   Surgery
   TENS unit
   Medications: Options for this: Antidepressants, anticonvulsants, NSIADS, Opioids, Period adjusting medications, Other:
   Hormonal treatments
   Bladder instillations

Psychological therapies (e.g., CBT, ACT, group therapy, counselling).
Sexual counselling/ sex therapy
Trigger point therapy
Pelvic floor manual therapy
Massage
Exercises provided by a physiotherapist
Electrotherapies (e.g., Ultrasound, Laser, TENS, Interferential etc)
Neuromodulation
Acupuncture
Exercise: please specify exercise type e.g., Yoga / Pilates/ Meditation/ Relaxation exercises etc
Education provided by health professionals on pelvic pain (e.g., likelihood of recovery, things you can do to recover)
None
Other Free text
For any treatment you selected did help, how did it help (free text)

2. Were you satisfied with the care provided by healthcare providers-?
   Yes/no

3. Did you feel fully informed about the treatment options you have received?
   Yes/ No

4. If no, what further information do you feel would have been important?
   Free text

5. Have you felt that you were part of the decision making regarding your care?
   Yes/ No

6. What things helped or would have helped you to feel in control of your care?
   Free text

7. Do you feel that the healthcare professionals you have seen listened to you about your pain?
   Yes/ No
   If your answer was no, what do you think would have improved the experience? (Free text)

8. Did you receive any information regarding your condition- that has helped you better manage/ understand your pain?
   Yes/ No
   If you selected yes, what information did you find most helpful? (Free text)

9. What concerns do you have about your condition in the longer term? (Free text)

10. Please chose a number that best reflects how confident you feel about the statement below.
    I feel I can manage my pain well
    0= not all confident, 1, 2,3, 4, 5,6, 7, 8,9, 10= completely confident

11. In general, I believe there is a pain treatment out there for me Y/N
    Please chose a number that best reflects how confident you feel about this statement.

0= not all confident, 1, 2,3, 4, 5,6, 7, 8,9, 10= completely confident

Treatment recommendations

1. What is important for you to understand about your condition? (Free text)

2. Who else in your life do you feel needs to understand more about your condition? (Free text)

3. What are the important issues that should be discussed as part of the treatment? (Free text)

4. How would you know if a treatment was effective? (Free text)

5. How would your life be different if your pain was better managed? (Free text)

If you would be interested intaking part in research in this area to register your interest, please contact: selina.johnson@nhs.net

Supplementary Materials 2 (S2): Modified STarTBacK tool.

**Original STarTBacK tool.**	**Modified pelvic version**
Q1. My back pain has spread down my leg(s) at some time in the last 2 weeks	Q1. I experience non-cyclical pelvic pain (pain at some time of the month, when not having a period)
Q2. I have had pain in the shoulder or neck at some time in the last 2 weeks	Q2. I have had pain in other parts of my body (not just my pelvis) at some time in the last month
Q3. I have only walked short distances because of my back pain	Q3. I have only walked short distances because of my pelvic pain
Q4. In the last 2 weeks, I have dressed more slowly than usual because of back pain	Q4. In the last month, I have dressed more slowly than usual because of pelvic pain
Q5. It's not really safe for a person with a condition like mine to be physically active	Q5. It's not really safe for a person with a condition like mine to be physically active
Q6. Worrying thoughts have been going through my mind a lot of the time	Q6. Worrying thoughts have been going through my mind a lot of the time
Q7. I feel that my back pain is terrible and it's never going to get any better	Q7. I feel that my pelvic pain is terrible and it's never going to get any better
Q8. In general, I have not enjoyed all the things I used to enjoy	Q8. In general, I have not enjoyed all the things I used to enjoy
Q9. Overall, how bothersome has your back pain been in the last 2 weeks?	Q9. Overall, how bothersome has your pelvic pain been in the last month?

Supplementary Materials 3 (S3)

Recruitment strategy.

- Identification of charities, support groups, professional bodies, and clinicians with specialist interest/ expertise in pelvic pain.
- Identified parties were provided with information regarding the study and a link to the study flyer.
- Identified parties and the study PI shared the flyer on social media channels.
- Identified parties were contacted every two to three weeks to further promote the survey and schedule further promotional posts.

**Identified charities and support groups**	**Professional bodies, healthcare agencies and executive members of these groups**
Pelvic Pain Support Network	The Pain Research Institute.
Wellbeing of women	Pelvic, Obstetrics and Gynaecology Physiotherapy
Pain relief foundation	Pain Physiotherapy Association
Vulva Pain Society	The Walton Centre NHS Trust
The brain charity	Council for Allied Health Professionals Research (CAHPR, national and Norwest consortium).
Fair treatment for women of Wales (FTWW).	Nursing, Midwives, and Allied Health Professionals (NMAHP) Cheshire and Merseyside
Endometriosis UK and regional branches.	
Pain concern	
Endometriosis cure	
Endometriosis support group (Ireland) @endo_group_Irl	

Supplementary materials 4 (S4): Adapted STarT tool classification subgroup assessment.

Table S4: Adapted STarT tool classification subgroup assessment illustrating the percentage of persons responding within the identified theme.

**Theme**	**Low STarT classification n=124**	**Med/ High STarT classification n=710**
**Understanding condition**		
Validation	**70%**	**68%**
Management	**23%**	**20%**
Treatment	**8%**	**11%**
**Future concerns**		
Worsening of symptoms	**23%**	**27%**
Key: n= number of people, %= percentage of persons responding within this theme		

Subgroup assessment of STarT classifications using broad theme titles was used to assess potential differences relating to future concerns and treatment desires. No major differences in the frequency of responses were observed between groups, however, the response language did differ. Evocative language was more commonly used to communicate distress by persons with med/high scores compared to more explicative language in persons scoring low.
